# Analysis of the progression of systolic blood pressure using imputation of missing phenotype values

**DOI:** 10.1186/1753-6561-8-S1-S83

**Published:** 2014-06-17

**Authors:** Tatsiana Vaitsiakhovich, Dmitriy Drichel, Marina Angisch, Tim Becker, Christine Herold, André Lacour

**Affiliations:** 1 Institute for Medical Biometry, Informatics and Epidemiology (IMBIE), University of Bonn, Sigmund-Freud-Str., D-53105 Bonn, Germany; 2 German Center for Neurodegenerative Diseases (DZNE), Ludwig-Erhard-Allee 2, D-53175 Bonn, Germany

## Abstract

We present a genome-wide association study of a quantitative trait, "progression of systolic blood pressure in time," in which 142 unrelated individuals of the Genetic Analysis Workshop 18 real genotype data were analyzed. Information on systolic blood pressure and other phenotypic covariates was missing at certain time points for a considerable part of the sample. We observed that the dropout process causing missingness is not independent of the initial systolic blood pressure; that is, the data is not missing completely at random. However, after the adjustment for age, the impact of systolic blood pressure on dropouts was no longer significant. Therefore, we decided to impute missing phenotype values by using information from individuals with complete phenotypic data. Progression of systolic blood pressure (∆*SBP*/∆*t*) was defined based on the imputed phenotypes and analyzed in a genome-wide fashion. We also conducted an exhaustive genome-wide search for interaction between single-nucleotide polymorphisms (7.14 × 10^10 ^tests) under an allelic model.

The suggested data imputation and the association analysis strategy proved to be valid in the sense that there was no evidence of genome-wide inflation or increased type I error in general. Furthermore, we detected 2 single-nucleotide polymorphisms (SNPs) that met the criterion for genome-wide significance (*p*≤5 × 10^−8^), which was also confirmed via Monte-Carlo simulation. In view of the rather small sample size, however, the results have to be followed-up in larger studies.

## Background

We present a genome-wide analysis, that is, an analysis comprising all odd-numbered chromosomes, of the Genetic Analysis Workshop 18 (GAW18) real data. The structure of the GAW18 data is rather complex (longitudinal study, family structure, missing measurements), making it difficult to address all dimensions of the data in a single analysis. Therefore, we restricted our analysis to 142 unrelated individuals available. Moreover, because systolic blood pressure (SBP) was analyzed extensively during the genome-wide association analysis era by large consortia, we decided to investigate the progression of SBP instead.

We address the problem of missing phenotypes and impute missing values for the original sample of 939 individuals. Based on the imputed phenotype data, we conduct a genome-wide analysis of the quantitative trait progression of SBP in time and perform an exhaustive search for SNP-SNP interaction. We assess the validity of the approach in the genome-wide setting and confirm our top-ranking findings via Monte-Carlo simulation. Finally, we compare results obtained with the imputed phenotypes to the respective results obtained with the original phenotype data.

## Methods

### Imputation of missing data in longitudinal studies

In a longitudinal study repeated measurements and records related to a trait under investigation are collected at different time points for a fixed group of individuals. It is often not possible to collect complete data sets because of early dropouts, changed health conditions of subjects, or other reasons. The first step in the analysis of repeated measurements with missing values should be the verification, whether the data is missing completely at random (MCAR), missing at random (MAR), or missing not at random (MNAR) [1]. An observation is said to be MCAR if the missingness is independent of all observed and unobserved assessments. A more relaxed assumption about the missing data mechanism is MAR, where missingness is independent of all unobserved values, although it may be dependent on the observed values. A process that is neither MCAR nor MAR is called MNAR [1-3]. Monotone missing data refers to data from a longitudinal study, where the subjects who left the study before it was finished never returned again.

Standard methods to treat missing data, for example, complete case analysis and simple imputation methods (last-observed-carried-forward, mean, etc), are not appropriate for a valid inference without assumption that the data is MCAR, or when this assumption is violated. A proper analysis of missing data requires a knowledge or estimate of the mechanism that generated the missing data [4]. A test for MCAR versus MAR can be found in Little [5]. Unfortunately, one cannot determine whether missingness is MNAR or MAR based solely on the given data [4].

In analyzing the GAW18 data we were dealing with quantitative, non-monotone data from the longitudinal study. For available phenotypic variables we introduced the following notations: *AGE_i _*for age, *SBP_i _*for SBP, *MED_i _*for intake of antihypertensive medication, and *SMK_i _*for smoking status at the initial measurement *i *= 0 and up to 3 follow-ups (*i *= 1,2,3).

We analyzed the quantitative trait progression of SBP in time, ∆*SBP/*∆*t*, which was defined as the difference between 2 values of SBP recorded at the last and the first examinations attended by an individual, divided by the time ∆*t *in years elapsed between these 2 examinations.

We suspected that the data was not MCAR and tried to determine whether MAR or MNAR was more proper to assume. We considered a linear regression model, *SBP*_0 _= β_0 _+ β_1_*x*_1 _+ β_2_*x*_2 _+ β_3_*x*_3_, where a variable *x_i _*equals 1 if the measure of *SBP_i _*is given, and 0 if the measure of *SBP_i _*is missing, β*_i_*∈ℝ, *i *= 0,1,2,3. Using this model to test the hypothesis β_1 _= β_2 _= β_3 _= 0 resulted in the *p*-value 5.21 × 10^−7^. This led us to conclude that the data is not MCAR, given that the presence or absence of the follow-up measures is influenced by *SBP*_0_. However, when we included the covariate *AGE*_0_, the *p*-value became much higher (*p *= 0.07), which suggested that age might be a significant predictor of missingness and that the data is likely MAR.

To impute the missing values and create complete phenotype data for each subject, we applied a kind of simple imputation method [2,3], which represents our modification of this well-known technique. For our purposes, we imputed the missing values in *AGE_i_, SBP_i_, MED_i_, SMK_i _*variables, *i *= 0,1,2,3, without considering hypertension status or diastolic blood pressure. To start the imputation we extracted 187 completers, that is, individuals who had participated in all 4 examinations and for whom all measurements had been recorded. We used the information collected for completers to determine the settings of the association analysis, as well as to complete the missing phenotype values for non-completers.

We suggest the following algorithm to impute the missing values in the GAW18 real phenotype data for the purposes of the analysis of ∆*SBP/*∆*t*:

1. Determine the average calendar years of the 4 examinations (here: 1993, 1998, 2003, 2009).

2. Impute the missing values of *AGE_i _, i *= 0,1,2,3, using the results of Step 1 and the birth year of every individual.

3. Calculate the correlation coefficients α_1 _, α_2 _, α_3 _of *∆SBP *on *AGE*_0_, *MED*_0_*, SMK*_0_, respectively, and β of *∆SBP *on *SBP*_0 _for completers.

4. Given a completer *C *and a non-completer *N*, define a function where wide hat denotes the normalized value of the corresponding variable for instance, and where *I_(·) _*equals 1 if the corresponding measurement for a non-completer is given, or equals 0 otherwise.

(1) $$\begin{array}{c} d\left(C,N\right):=\sum_{i=0}^{3}[\left|\frac{\alpha_{1}}{\beta }\right|I_{AGE_{i}^{N}}{\left({\hat{AGE}}_{i}^{N}-{\hat{AGE}}_{i}^{C}\right)}^{2}+I_{SBP_{i}^{N}}{\left({\hat{SBP}}_{i}^{N}-{\hat{SBP}}_{i}^{C}\right)}^{2}+\\ \left|\frac{\alpha_{2}}{\beta }\right|I_{MED_{i}^{N}}{\left({\hat{MED}}_{i}^{N}-{\hat{MED}}_{i}^{C}\right)}^{2}+\left|\frac{\alpha_{3}}{\beta }\right|I_{SMK_{i}^{N}}{\left({\hat{SMK}}_{i}^{N}-{\hat{SMK}}_{i}^{C}\right)}^{2}] \end{array}$$

(2) $${\hat{AGE}}_{i}^{N}=\frac{AGE_{i}^{N}-\mu_{i}^{N}}{\sigma_{i}^{N}},\mu_{i}^{N}=\sum_{N}\frac{AGE_{i}^{N}}{N},\sigma_{i}^{N}=\sqrt{{\sum_{N}\left(AGE_{i}^{N}\right)}^{2}-{\left(\mu_{i}^{N}\right)}^{2}}$$

5. For every fixed non-completer determine the "closest" completer by finding the minimal value of the function *d *on the list of all completers.

6. Impute the missing values of *SBP_i_, MED_i _, SMK_i _, i *= 0,1,2,3, for a given non-completer by those from the "closest" completer.

Imputation was performed for 752 non-completers. Table 1 provides an example of imputation.

**Table 1:** Imputation of the missing phenotype data

ID	*N*/*C*	*d*(*C,N*)	*AGE_0_*	*AGE_1_*	*AGE_2_*	*AGE_3_*	*SBP_0_*	*SBP_1_*	*SBP_2_*	*SBP_3_*	*MED_0_*	*MED_1_*	*MED_2_*	*MED_3_*	*SMK_0_*	*SMK_1_*	*SMK_2_*	*SMK_3_*
T2DG2501049	N		**39**	45	50	**56**	**110**	125	139	**149**	**0**	0	0	**1**	**0**	0	0	**0**

T2DG0300150	C	0.072	35	41	45	51	110	121	135	149	0	0	0	1	0	0	0	0
T2DG1700856	C	0.108	33	38	43	50	110	123	130	132	0	0	0	1	0	0	0	0
T2DG0600470	C	0.268	43	46	51	58	133	127	131	185	0	0	0	0	1	1	0	1
...				...				...				...				...		
T2DG0300165	C	18.105	50	57	61	66	151	175	176	139	0	0	0	1	0	0	0	0
T2DG0600450	C	21.673	48	53	61	65	136	190	128	130	0	1	1	1	1	0	0	0

The results of imputation are shown for a middle-aged non-completer (*N*) with ID number T2DG2501049 (see GAW18 real phenotype data) with 2 missing values in each phenotypic variable. The real SBP measurements indicate the possible development of hypertension by this individual. In the table, several values of the distance-function *d(C,N) *are shown in ascending order. The completer (*C*) T2DG0300150 is closest to the given non-completer. The imputed values are shown in bold.

### Quality control (GAW18 real genotype data)

First, we filtered 142 individuals from the GAW18 data set who were indicated as being nonrelated. To check for unobserved relatedness among these individuals, we computed a pair-wise identity-by-state (IBS) matrix from the genotype data. For a pair of individuals, their IBS-value was computed as the portion of alleles identical by state divided by the number of alleles genotyped in both individuals. The mean IBS-value over all pairs of individuals was 0.74; its SD (standard deviation) was 0.0063. Seven pairs had IBS-values higher than 0.84, which was more than 14 SD above the mean, indicating evidence of relatedness. For each of the 7 pairs, the individual with the lower genotyping rate was removed from the analysis. We also excluded 8 other individuals who had genotype missing rates exceeding 40%, leaving 127 individuals for analysis.

Finally, we removed 42,519 SNPs because of either low genotyping rate (<99%), deviations from Hardy-Weinberg equilibrium (*p*<1 × 10^−6^), or low minor allele frequency (MAF<1%). That left 429,516 quality controlled SNPs for analysis, and the overall genotyping rate of the remaining data was 99.9%.

### Statistical analysis

For the association analysis of ∆*SBP*/∆*t *reasonable covariates would be *AGE*_0_*, *SBP*_0_*, *MED*_0_*, *SMK*_0_*, ∆*MED*, ∆*SMK*, and *SEX*. Here, for every individual, the variables *AGE*_0_*, *SBP*_0_*, *MED*_0_*, *SMK*_0_*are defined to be equal to the first non-missing *AGE_i _, SBP_i_, MED_i _, SMK_i _*values, respectively; *i *= 0,1,2,3; ∆*MED *and ∆*SMK *are defined analogously to ∆*SBP*. Note that when using imputed phenotype data, *AGE*_0_*= *AGE*_0 _, *SBP*_0_*=*SBP*_0_, etc. From the plausible covariates listed above, we retained for the association analysis only those covariates that were significantly associated with ∆*SBP*/∆*t *in the sample of completers: *AGE*_0_*, *SBP*_0_*, *MED*_0_*, and *SEX*.

For single-marker analysis, we applied the standard linear regression test with 1 degree of freedom (DF) for quantitative traits and used the implementation in INTERSNP [6].

For 2-marker analysis, we used a test for allelic interaction. We compared the sum of standard errors (SSE) of the model *M^A,I^*= β_0 _+ β_1_*x*_1 _+ β_2_*x*_2 _+ β_1,2_*x*_1_*x*_2 _against the model *M ^A ^*= β_0 _+ β_1_*x*_1 _+ β_2_*x*_2_, where β_0 _is the intercept parameter *x_i _*is the number of copies of minor alleles of SNP *i, i *= 1,2; for a given individual, *x*_1_*x*_2 _is the interaction term; and βs are the coefficients to be estimated. Let *SSE^A,I ^*and *SSE^A ^*be SSEs of the respective models, and let *n *be the number of individuals in the analysis. Then, the test statistic *t *= (*SSE^A ^*− *SSE^A,I^*)/*SSE^A ^*is *F*-distributed with (*n *− 4, 1) DF and yields a test for allelic interaction. Analysis was performed by a parallelized version of INTERSNP, which uses the OpenMP API specification for parallel programming (http://openmp.org/wp). In the interaction analysis, we applied a stronger MAF criterion of 5% and analyzed 7.14 × 10^10 ^SNP pairs.

When the sample size is small, low MAF can lead to a small number of "effective" analysis observations and the application of the *F*-distribution might lead to inflated *p*-values. To overcome this issue, we validated the top-ranking *p*-values of the single-marker analysis via Monte-Carlo simulation. In each permutation replicate, the quantitative trait values were randomly distributed over the individuals of the sample. In total, 10^8 ^permutation replicates were conducted and for a given SNP, its point-wise Monte-Carlo *p*-value was computed as *k*/10^8^, where *k *is the number of permutation replicates with a test statistic *t *larger than that of the real data. For computation time reasons, it was not possible to validate the results of the interaction analysis via simulation, since at least 10^13 ^replicates would have been needed.

## Results

The analysis of ∆*SBP*/∆*t *yielded a genome-wide inflation factor of λ = 0.979, indicating that, overall, our analysis can be judged as valid in the sense that we do not have evidence for inflated *p*-values caused either by population stratification or by our phenotype imputation method. Table 2 lists the best results of the single-marker analysis. Two SNPs, rs3093642 (*p *= 1.35 × 10^−9^) and rs13157168 (*p *= 2.45 × 10^−8^), met the criterion for genome-wide significance. These SNPs have rather low MAF, and the results might be artifacts. However, Monte-Carlo simulation with 10^8 ^permutation replicates confirmed the findings; none of the replicates had a more extreme test statistic than the real data. Nevertheless, the top SNP rs3093642 should be treated with caution because its *p*-value for deviation from Hardy-Weinberg equilibrium was rather close to the predefined exclusion criterion. The second SNP rs13157168 is an intronic variant in the *RASA1 *gene. Association of *RASA1 *with variable capillary and arteriovenous malformations was reported by Boon et al [7]. Note that the *p*-values *p_r _*of the single-marker analysis performed on SNPs on the *real phenotypes *are markedly higher than the corresponding *p*-values *p_i _*for the imputed phenotypes. SNPs with an at least suggestive level of evidence for association, unfortunately, were not found among the top ranks.

**Table 2:** Single-marker analysis of ∆*SBP*/∆*t *on unrelated individuals

SNP	Position	Alleles	MAF	HWE	β ± σ	*p_i_*	*p_i_^MC^*	*p_r_*	*p_r_^MC^*
rs3093642	1-25687742	T/C	0.02	1.23e-5*	2.68 ± 0.41	1.35e-9	0†	0.57	0.49
rs13157168	5-86627138	A/G	0.01	0.893	3.83 ± 0.64	2.45e-8	0†	0.48	0.41
rs956918	9-436983	C/A	0.05	0.002	1.54 ± 0.29	4.14e-7	4.00e-7	0.04	0.05
rs6960510	7-147977001	G/A	0.04	0.641	1.91 ± 0.37	1.32e-6	1.60e-6	0.27	0.26
rs7120076	11-111404684	G/A	0.02	1.23e-5*	2.17 ± 0.43	1.38e-6	1.00e-7	0.46	0.39
rs11803060	1-76549233	C/T	0.04	0.644	1.82 ± 0.37	3.14e-6	3.10e-6	0.40	0.39
rs11665668	19-52169702	A/G	0.04	0.029	1.69 ± 0.35	3.47e-6	3.10e-6	0.09	0.08
rs1340503	9-78571504	C/T	0.20	0.471	0.90 ± 0.19	3.78e-6	4.10e-6	0.09	0.09
rs6860971	5-88552414	T/C	0.04	0.103	1.52 ± 0.32	7.18e-6	8.90e-6	0.36	0.34
rs1202389	7-148913645	T/G	0.06	0.449	1.43 ± 0.31	7.46e-6	9.80e-6	0.49	0.48
rs10438410	15-92055287	T/C	0.03	0.749	2.07 ± 0.44	7.61e-6	9.30e-6	0.44	0.40
rs1524846	15-24025870	G/T	0.02	0.785	2.25 ± 0.48	8.18e-6	8.80e-6	0.01	0.02
rs12269879	11-108413351	A/G	0.02	0.857	2.67 ± 0.58	8.91e-6	9.70e-6	0.25	0.22

*Indicates *p*-values for Hardy-Weinberg equilibrium close to the exclusion criterion.

†None of 10^8 ^permutation replicates had a more extreme test statistic than the real data.

The best results of the single-marker analysis are shown with SNP ID; physical base-pair position; minor/major alleles, MAF; Hardy-Weinberg equilibrium (HWE) *p-*value; regression coefficient β with standard deviation σ; *p-*value *p_i _*of the single-marker analysis performed on the imputed phenotypes; *p-*value of the Monte-Carlo simulation *p_i_^MC ^*corresponding to *p_i_*; *p-*value *p_r _*of the single-marker analysis performed on SNPs listed in the table and on real phenotypes; and *p-*value of the Monte-Carlo simulation *p_r_^MC ^*corresponding to *p_r_*.

Table 3 presents the top results of the interaction analysis. 2 SNPs from the table, rs4686358 and rs7987982, were previously reported in connection with potentially related phenotypes. The variant rs4686358 is located close to rs35964523 (≈130 kilobases [kb]), a SNP that was implicated in total cholesterol levels (*p *= 7.9 × 10^−6^) [8]. SNP rs7987982 is an intronic variant of the *COL4A1 *gene. Variants in *COL4A1 *are significantly associated with brain small vessel disease, in which stiffness of blood vessels is affected [9].

**Table 3:** Two-marker interaction analysis of ∆*SBP*/∆t on unrelated individuals

SNP_1_						SNP_2_								
				
rs	Position	Alleles	MAF	HWE	*p_i_*	rs	Position	Alleles	MAF	HWE	*p_i_*	β ± σ	*p_i_^INT^*	*p_r_^INT^*
rs4686358	3-1028349	T/C	0.15	0.419	0.52	rs17236800	7-37944375	G/A	0.09	0.053	0.19	3.27 ± 0.39	1.20e-13	0.157
rs1321632	3-197092657	T/C	0.06	0.456	0.01	rs7987982	13-110931311	C/T	0.09	0.263	0.22	1.73 ± 0.21	1.24e-13	0.395
rs12632248	3-185139913	A/G	0.10	0.518	0.13	rs10770039	11-9587187	T/C	0.28	0.017	0.56	2.97 ± 0.36	2.78e-13	0.503
rs1482590	3-65219095	T/C	0.20	0.361	0.09	rs10488631	7-128594183	C/T	0.23	0.754	0.22	1.72 ± 0.21	6.82e-13	0.598
rs11716921	3-17175592	G/T	0.17	0.104	0.11	rs6870951	5-133064960	T/C	0.06	0.511	0.43	4.06 ± 0.51	1.45e-12	0.086
rs2220368	5-176140452	A/G	0.21	0.695	0.92	rs7810996	7-73624615	T/C	0.31	0.511	0.78	2.85 ± 0.37	3.48e-12	0.081
rs249209	5-79867209	C/A	0.25	0.069	0.34	rs427488	17-12428237	C/T	0.11	0.163	0.18	2.51 ± 0.32	3.62e-12	0.416
rs7719329	5-108391660	T/C	0.09	0.053	0.13	rs4868699	5-176134402	C/T	0.22	0.222	0.02	2.04 ± 0.27	4.45e-12	0.013
rs524138	5-31465137	A/G	0.07	0.417	0.06	rs4885678	13-80440491	T/G	0.22	0.981	0.50	3.41 ± 0.45	5.90e-12	0.048
rs17242130	5-81154906	C/A	0.06	0.517	0.32	rs2836412	21-39825012	C/T	0.25	0.617	0.08	3.18 ± 0.42	6.38e-12	0.665
rs1677694	5-79936297	T/C	0.17	7.19e-4*	0.23	rs12673145	7-81423463	G/T	0.11	0.679	0.16	2.91 ± 0.38	7.07e-12	0.027
rs3757572	7-45146656	C/A	0.27	0.552	0.55	rs7275197	21-39317417	G/A	0.14	0.235	0.25	3.70 ± 0.49	8.58e-12	0.137
rs1802074	7-37947103	A/G	0.13	0.579	0.50	rs3887013	15-93614127	T/C	0.09	0.240	0.57	3.92 ± 0.52	9.41e-12	0.043

* Indicates *p*-values for Hardy-Weinberg equilibrium close to the exclusion criterion.

The best results of the 2-marker interaction analysis are shown. Each line contains a pair of SNPs with their IDs; physical base positions; minor/major alleles; Hardy-Weinberg equilibriums (HWEs); *p-*values *p_i _*of the single-marker analysis performed on the imputed phenotypes; regression coefficient β with standard deviation σ of the interaction analysis; *p-*value *p_i_^INT ^*of the interaction analysis performed on the imputed phenotypes; and *p-*value *p_r_^INT ^*of the interaction analysis performed on SNPs listed in the table and on the real phenotypes.

## Discussion

In longitudinal studies, repeated measurements related to a trait under investigation are performed subsequently within a particular time period. The attractiveness of longitudinal studies is the ability to capture the dynamics of traits. Investigating the progression of disease by using this additional dimension of time might reveal insights on underlying disease mechanisms. Given the nature and the running time of a longitudinal study, it is expected that some measurements might not be available, whereas others might be missed. Imputation of missing values helps to enrich data and to use maximum information, although it has own weaknesses and it is not self-evident that it leads to improved power.

In our setting, the imputation of missing values in the GAW18 phenotype data set helped to streamline the definition of the trait under investigation (∆*SBP*/∆*t*). In particular, the imputation reduced the impact of outlier SBP values, and made it possible to define the values of the trait in a more uniform time period. This, in turn, helped to reduce the potentially negative impact of short time periods on the values of the trait.

The method of choice to check the validity of imputation is to carry out a sensitivity analysis to investigate the dependence of the results on the particular imputation method. In our case, the sensitivity analysis could have been carried out by the multiple imputation technique. Unfortunately, this technique is time-consuming and requires software implementation; consequently, it was not applied in our work. This is a potential limitation of our approach. An argument in favor of our imputation method is that the mean of SBP values within an examination is preserved by the imputation.

## Conclusions

Our analysis strategy helped to identify some suggestive association findings. At this point, however, the results do not prove that the analysis using imputed phenotype data is superior to that without imputation. To prove this statement, it would be necessary to know whether some of the SNPs we have identified represent "true" associations. Those have to be investigated in studies with independent data. In summary, our work can be seen as a suggestion for longitudinal data analysis, which was quite reasonable in the sense that it did not show genome-wide inflation or increased type I error in general.

## Competing interests

The authors declare that they have no competing interests.

## Authors' contributions

TV designed and performed the imputation of the missing phenotypes. TV and DD carried out the analysis of unrelated individuals. MA worked on the pre-processing of the data. CH provided support in using INTERSNP software. TB conceived of the study and suggested main lines of the analysis. AL coordinated the study, participated in its design and improvement. All authors read and approved the final manuscript.
